# Mutation in the Kv3.3 Voltage-Gated Potassium Channel Causing Spinocerebellar Ataxia 13 Disrupts Sound-Localization Mechanisms

**DOI:** 10.1371/journal.pone.0076749

**Published:** 2013-10-07

**Authors:** John C. Middlebrooks, Harry S. Nick, S. H. Subramony, Joel Advincula, Raymond L. Rosales, Lillian V. Lee, Tetsuo Ashizawa, Michael F. Waters

**Affiliations:** 1 Departments of Otolaryngology, Neurobiology & Behavior, Cognitive Sciences and Biomedical Engineering and Center for Hearing Research, University of California Irvine, Irvine, California, United States of America; 2 Department of Neuroscience, McKnight Brain Institute, University of Florida College of Medicine, Gainesville, Florida, United States of America; 3 Departments of Neuroscience and Neurology, McKnight Brain Institute, University of Florida College of Medicine, Gainesville, Florida, United States of America; 4 Western Visayas State University Medical Center, Iloilo City, Philippines; 5 Department of Neurology and Psychiatry, University of Santo Tomas, Manila, Philippines; 6 Child Neuroscience Center, Philippine Children’s Medical Center, Quezon City, Philippines; University of Houston, United States of America

## Abstract

Normal sound localization requires precise comparisons of sound timing and pressure levels between the two ears. The primary localization cues are interaural time differences, ITD, and interaural level differences, ILD. Voltage-gated potassium channels, including Kv3.3, are highly expressed in the auditory brainstem and are thought to underlie the exquisite temporal precision and rapid spike rates that characterize brainstem binaural pathways. An autosomal dominant mutation in the gene encoding Kv3.3 has been demonstrated in a large Filipino kindred manifesting as spinocerebellar ataxia type 13 (SCA13). This kindred provides a rare opportunity to test *in vivo* the importance of a specific channel subunit for human hearing. Here, we demonstrate psychophysically that individuals with the mutant allele exhibit profound deficits in both ITD and ILD sensitivity, despite showing no obvious impairment in pure-tone sensitivity with either ear. Surprisingly, several individuals exhibited the auditory deficits even though they were pre-symptomatic for SCA13. We would expect that impairments of binaural processing as great as those observed in this family would result in prominent deficits in localization of sound sources and in loss of the "spatial release from masking" that aids in understanding speech in the presence of competing sounds.

## Introduction

The auditory system is distinguished by the great precision with which it analyses temporal and intensive features of sounds. Well practiced human listeners can detect ITDs as brief as ~10 µs [1,2] and ILDs as low as ~1 dB [3]. This fine acuity has been attributed in part to the rapid and precisely timed responses of voltage-gated potassium channels expressed throughout the auditory brainstem [4,5,6]. Of particular relevance to the present study, the Kv3.3 (KCNC3) *Shaw* subtype is present at high levels in the medial nucleus of the trapezoid body (MNTB) [4,5], which is a key element in binaural pathways. Channels in the Kv3 subfamily function during the peak of the action potential to hasten repolarization, enabling rapid neuronal firing [7,8].

Autosomal dominant mutations in the gene encoding Kv3.3 have been demonstrated in both French [9] and Filipino kindreds [10,11]. The two distinct mutations result in differing biophysical channel properties and clinical phenotypes, though both are causative for SCA13. In the Filipino family, affected individuals exhibit an adult onset of symptoms that include prominent gait and limb ataxia and cerebellar atrophy [10,11,12]. The mutation in this family, *KCNC3* ^R420H^, is located on the S4 trans-membrane domain of the channel, which functions as the main voltage-sensing element [12]. Expression of the mutant allele in *Xenopus laevis* oocytes reveals an absence of channel activity, and co-expression of the mutant with the wild-type gene results in dominant-negative suppression of conductance [12].

The high levels of Kv3.3 expression in the auditory brainstem suggest that a mutation in the corresponding gene may result in disruption of central auditory processing. Specifically, one might expect to find disorders of binaural hearing, which involves the anterior ventral cochlear nucleus (AVCN), the MNTB, the medial superior olive (MSO), and the lateral superior olive (LSO). We tested this hypothesis by evaluating 13 mutation-carrying members of the Filipino family for sensitivity to ITDs and ILDs. Control groups were 6 homozygote wild-type family members and 16 age-matched non-familial normal-hearing listeners. Most of the individuals with the defective gene showed profound deficits in ITD and ILD sensitivity, despite showing no obvious loss of pure-tone sensitivity in either ear. The binaural hearing deficit was uncorrelated with the presence or absence of SCA13 cerebellar ataxia symptoms. The present results demonstrate the importance of a particular ion channel component for a key aspect of human hearing.

## Materials and Methods

### Ethics Statement

This research protocol was approved by the University of Florida Institutional Review Board (UF IRB), and written informed consents were obtained and remain on file for all study participants including minors, in which case written consents were obtained from both the individuals and their parents in accordance with the UF IRB.

At the time of the study, the Filipino pedigree consisted of the female proband, her 16 offspring (generation II, 12 living), and their children (generation III) and grand-children (generation IV) [11]. We studied 19 members of generations II, III, and IV, 13 of whom were heterozygous for the mutation ("affected listeners") and 6 of whom lacked the mutation ("familial controls"). The 19 family members ranged in age from 10 to 88 yrs (one 10 yr and all others 20-88 yr; median 42 yr, 12 female). Hearing status of members of the family was tested without prior knowledge of their genotype. We also tested 16 non-related normal-hearing age-matched individuals ("non-familial controls") who were recruited from the laboratory and clinical staff at the University of Florida. The non-familial controls ranged in age from 7 to 57 yr (one 7 yr, one 9 yr, and all others 25-57 yr; median 35 yr, 15 female). The distributions of ages did not differ significantly among the three groups (Kruskal-Wallis χ^2^_(2,32)_ = 1.52, *p* = 0.47).

**Figure 1:**
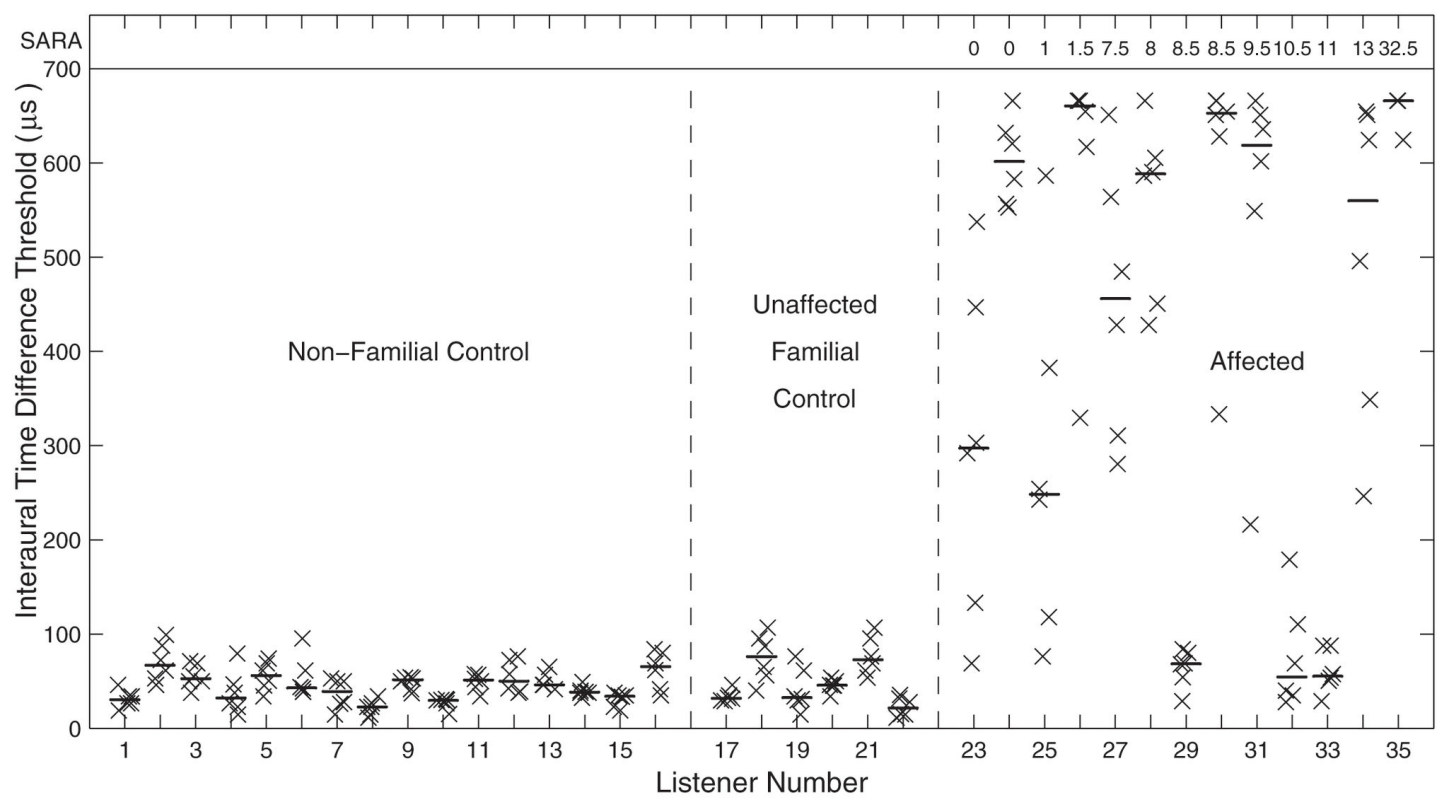
Interaural-time-difference (ITD) thresholds. Groups of listeners are sorted by non-familial control, unaffected familial control, and affected groups, and the affected listeners are sorted by their SARA scores, which indicate degree of cerebellar ataxia as described in the text. SARA scores for affected listeners are given as the row of numbers at the top of the figure. Each vertical column of X's represents ITD thresholds for one listener measured using a 3-down-1-up adaptive procedure. The horizontal line segment in each column indicates the median value for that listener. Horizontal positions of symbols are jittered to improve readability.

**Figure 2:**
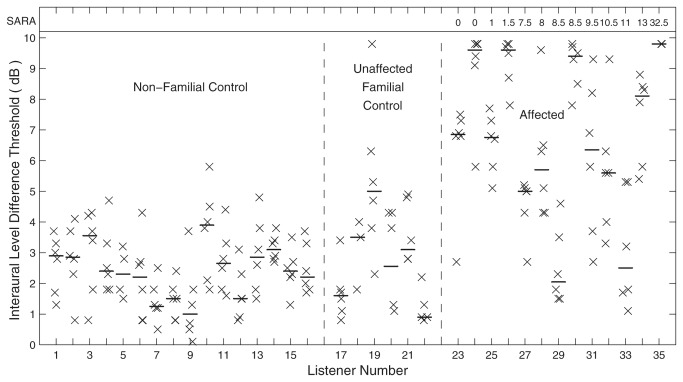
Interaural-level-difference (ILD) thresholds. Each vertical column of X's represents ILD thresholds for one listener measured using a 3-down-1-up adaptive procedure. The horizontal line segment in each column indicates the median value for that listener. All other conventions are as in Figure 1.

Genotype status of all affected and unaffected Filipino family members was confirmed by complete forward and reverse strand sequencing of the *Kv3.3* gene. That the mutation existed in isolation without additional gene variation was verified by analyzing the full coding sequence as well as intron-exon boundaries. In each affected individual, sequencing revealed the c.1259GA mutation giving rise to p.Arg420His. This was absent in all unaffected listeners. Affected and unaffected family members were evaluated for clinical signs of cerebellar ataxia. Those results for each individual were summarized by the Scale for the Assessment and Rating of Ataxia (SARA), which can range from 0 (asymptomatic) to 40 (severe disability) [13]. A value of 5 or higher indicates clear signs observable on routine clinical neurological exam. All of the unaffected individuals received SARA scores of 0. Among the individuals shown by molecular testing to carry the mutated gene, scores ranged from 0 to 32.5; the SARA scores are given in figures 1 and 2.

Hearing tests for 10 of the affected and all of the non-affected family members were conducted at sites in the Philippines that were convenient for those listeners, including a variety of conference rooms, hotel rooms, apartments, etc; the 3 remaining family members were tested in an office in Los Angeles. The non-familial listeners were tested in home or office settings in Florida. Pure-tone audiometry was performed using an Ambco model 650AB audiometer. Tested frequencies were 0.25, 0.5, 1, 2, 3, 4, 6, and 8 kHz. Right- and left-ear audiograms were obtained from 10 of the 13 affected listeners and 5 of the 6 unaffected family members. Audiometric booths were not used for any of the tests. The presence of ambient noise in these conditions resulted in elevation of pure-tone detection thresholds by ~20-30 dB, particularly at low frequencies. Nevertheless, as shown in the Results, there were no significant differences in pure-tone audiograms between affected and non-affected family members, and ITD and ILD thresholds for control listeners overlapped with published values. For those reasons, we conclude that the non-standard testing venues had no serious impact on the goals of this study.

Psychophysical tests of ITD and ILD sensitivity were conducted using a MacBook Pro laptop computer (Apple, Inc., Cupertino, CA) with its default built-in sound card. Sounds were presented dichotically through Sennheiser model HD280 Pro closed circumaural headphones (Old Lyme, CN). Stimulus presentation and data acquisition employed custom MATLAB scripts (The Mathworks, Natick, MA), including a graphical user interface.

All sound stimuli for the binaural tests were bursts of independent (i.e., non-frozen) Gaussian noise, 10 ms in duration plus 1-ms rise and fall times. For the tests of ITD sensitivity, the Gaussian noise bursts were low-pass filtered at 1600 Hz using a second-order Butterworth filter implemented in the MATLAB script. The low frequencies were intended to emphasize the contributions of pathways involving the medial superior olive (MSO), which has been shown in animals to contain primarily neurons tuned to low frequencies [14]. Interaural delays were introduced in the entire waveform, including fine structure and envelope, although we assume that at those low frequencies listeners would be more sensitive to delays in the fine structure than in the envelope. For the ILD tests, the Gaussian noise bursts were high-pass filtered at 3000 Hz, also using a second-order Butterworth filter. The high frequencies were intended to emphasize the pathway involving the lateral superior olive (LSO), which is regarded as a high-frequency nucleus [14]. Interaural differences of *L* dB were implemented by increasing the sound level by L/2 dB at one ear and decreasing the level by L/2 dB at the other ear. The ILDs were 0 dB during ITD testing and ITDs were 0 µs during ILD testing.

The filtered noise bursts were directed to the two ears, with or without introduction of an interaural delay or level difference. At the beginning of each test session, the listener clicked on a button on the computer screen, which triggered a 10-ms low- or high-passed noise burst. The listener was instructed to repeatedly click that button and to adjust the laptop volume controls until the sound was "clear and comfortable". When satisfied with the presentation level, the listener clicked a button to begin a series of trials.

Tests of ITD and ILD sensitivity used essentially the same two-interval, three-down-one-up adaptive tracking procedure. The listener began each trial by clicking on a "Go" button. After a brief delay, a noise burst was presented diotically (i.e., with no interaural difference), followed after 700 ms by a burst presented dichotically. The listener was instructed to click on "Left" or "Right" buttons to indicate whether the second sound appeared to the left or right of the first, diotic, sound. That procedure was repeated, following the three-down-one-up rule for adjusting ITD or ILD, tracking 79.4% correct [15]. Adjustments in ILD were 2 dB initially, reducing to 0.5 dB. Changes in ITD were 50% initially, reducing to 10%. The ranges of ITDs and ILDs were limited to ±700 µs and ±10 dB, respectively. Each series of trials continued until there were 10 reversals in the track, and then threshold ITD or ILD was given by the mean of the last 6 reversals. Six such estimates of ITD and ILD threshold were obtained for nearly all the listeners. The exceptions were one of the affected listeners, who completed only 3 ITD series and 2 ILD series, one familial control, who completed 6 ITD but only 3 ILD series, and one non-familial control, who completed 6 ITD and only 4 ILD series.

Data analysis used custom MATLAB scripts employing the MATLAB Statistics Toolbox. Multiple comparisons were corrected using Tukey’s least significant difference procedure.

## Results

The affected family members showed essentially normal left- and right-ear sensitivity to pure tones. Pure-tone audiogram levels at individual frequencies showed sizeable variance among listeners (standard deviations of 10.0 to 16.4 dB, depending on frequency), consistent with the wide range of ages tested. None of the family members reported hearing disabilities or hearing-aid use. With few exceptions, distributions of hearing levels seen for affected and unaffected family members overlapped. We computed pure tone averages at low (0.25, 0.5, 1, and 2 kHz) and high (3, 4, 6, and 8 kHz) frequencies covering the ranges used in the ITD and ILD testing, respectively. There were no significant differences between affected and unaffected groups either at low frequencies (*p* = 0.60, Kolmogorov-Smirnov statistic = 0.30, *N* = 14 subjects x 2 ears) or high frequencies (*p* = 0.81, Kolmogorov-Smirnov statistic = 0.25).

Most of the listeners that tested positive for the mutation showed striking elevations in ITD thresholds compared to their unaffected relatives and age-matched non-related controls. In Figure 1, listeners are ordered by non-familial control, unaffected familial control, and affected groups with the affected listeners further ordered by their SARA scores. Analysis of variance (ANOVA) showed a significant dependence of listeners’ median ITD thresholds on group (*F*_(2,32)_ = 25.78*, p* < 10^-6^). Median thresholds of affected listeners were significantly higher than those of either control group (*p* < 0.001, corrected paired comparison), whereas there was no significant difference between median values of familial and non-familial controls (*p* > 0.05). The median ITD thresholds of the two control groups formed a homogeneous distribution, with mean and standard deviation of 45.0 ± 15.6 µs. These values correspond well with published thresholds of untrained normal-hearing listeners [16]. The ITD thresholds of the affected listeners were more heterogeneous. All but three had ITD thresholds significantly higher than those of any control listeners (*p* < 0.05, corrected paired comparisons). The majority of those listeners had median ITD thresholds ≥ 500 µs, which approaches the maximum value that was tested and the maximum value produced by free-field sounds. The remaining 3 listeners (numbers 29, 32, and 33, 2 male, 1 female) showed median ITD thresholds of 68, 55, and 56 µs, which were within the control range.

Similar patterns of deficits were observed for ILD thresholds (Figure 2). Median ILD thresholds varied significantly with group (*F*_(2,32)_ = 23.11*, p* < 10^-6^), thresholds of the affected group were significantly higher than those of either control group (*p* < 0.001, corrected paired comparison), and the two control groups were not significantly different from each other (*p* > 0.05). The median ILD thresholds of the two control groups averaged 2.5 ± 1.0 dB, again similar to published thresholds for untrained listeners [16]. Two of the affected listeners (29 and 33) had median ILD thresholds of 2.1 and 2.5 dB, but all other affected listeners had median ILDs of 5.0 dB or greater.

**Figure 3:**
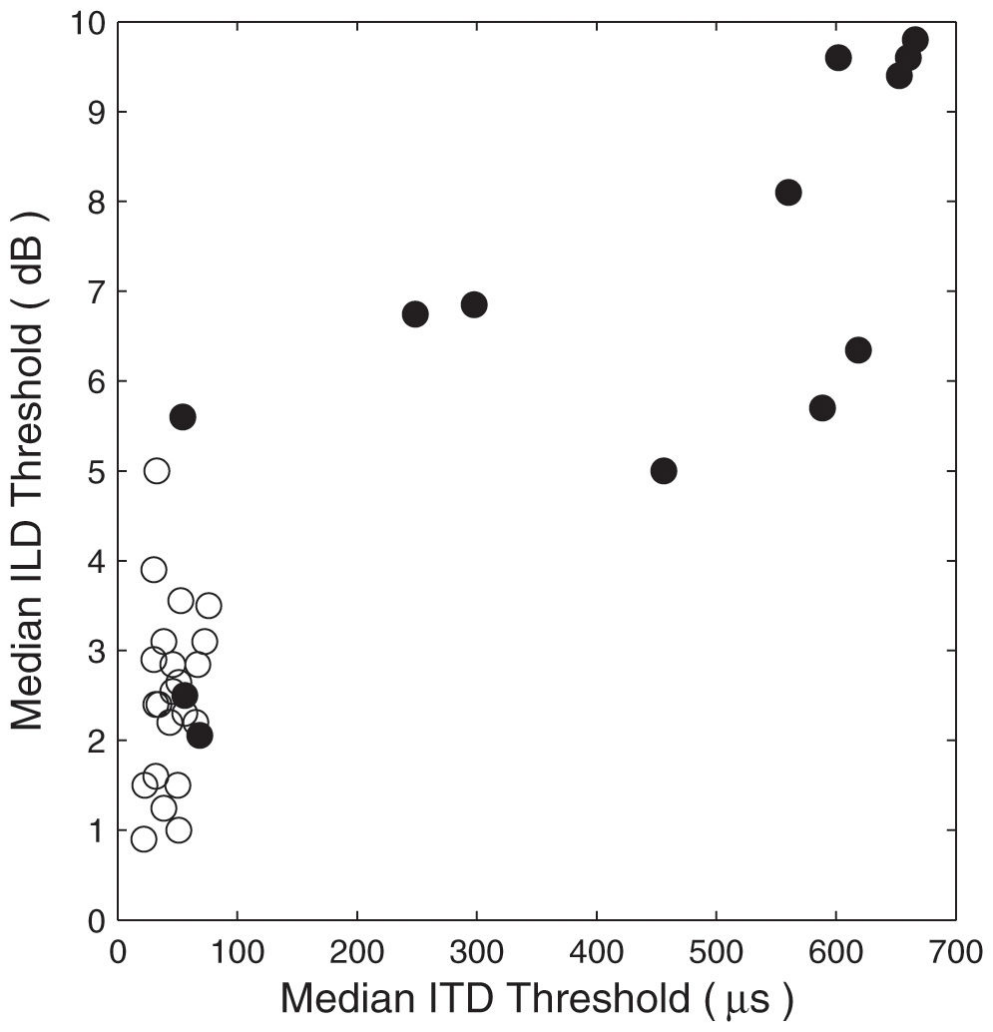
Relationship of ILD and ITD sensitivity. Each symbol represents data from one listener, with median ILD thresholds and median ITD thresholds plotted in the vertical and horizontal dimensions, respectively. Open circles represent non-familial and unaffected familial control listeners, and filled symbols represent affected listeners.

Among the affected listeners, median ILD thresholds correlated significantly with median ITD thresholds (*N* = 13, Pearson ρ = 0.79, *p* = 0.0013). The relation is plotted in Figure 3, with data from the affected listeners indicated with filled symbols. Two of the affected listeners (29 and 33) showed both ITD and ILD thresholds within the distribution of data from the control groups, and a third affected listener (32) had an ITD threshold in the control range and an ILD threshold in the lower part of the affected-listener distribution.

Surprisingly, no systematic relationship was observed between the severity of ataxia represented by the SARA scores and the ITD or ILD sensitivity (*N* = 13, Pearson ρ = 0.16, *p* = 0.60 for ITD; ρ = 0.09, *p* = 0.77 for ILD). The three affected listeners with the lowest (i.e., best) ITD thresholds had SARA scores ranging from 8.5 to 11, indicating clear neurological signs, whereas four listeners having SARA scores from 0 to 1.5 had moderate (listeners 23 and 25) or high (listeners 24 and 26) ITD thresholds. The lack of significant correlation between auditory and cerebellar disability suggests that expression of the mutant allele, and possibly *Shaw* subtype selection, may be regulated differently in auditory and cerebellar pathways. One example of such local regulation of Kv3.3 expression has been demonstrated in a study of the MNTB in organotypic culture showing that Kv3.3 expression increases under conditions of chronic depolarization [17]. The present results indicate that an auditory deficit could reveal the mutation even in individuals not yet showing overt postural and motor compromise. Indeed, one of the affected listeners had ITD and ILD thresholds that were among the worst in the sample and showed mild cerebellar atrophy [11]. Nevertheless, that listener showed no signs of ataxia or cerebellar pathology on clinical exam, consistent with being younger than the 25-to-60-yr age at onset observed in other clinically affected family members [10,11].

## Discussion

The present results demonstrate that a mutation of the gene encoding the Kv3.3 voltage-gated potassium channel can disrupt binaural hearing in humans. In rodents, Kv3.3 channels are found on bushy cells of the AVCN, in the LSO, in the MSO, and in particularly high levels in the MNTB [4,5,6]. Principal cells of the MNTB receive projections from globular bushy cells in the contralateral AVCN terminating on each cell in a giant synapse, the calyx of Held. The multiple release sites in the calyces can drive the MNTB cells at high spike rates with great temporal precision. Those MNTB cells, in turn, send sustained stimulus-locked inhibition to the MSO and LSO in the form of somatic glycinergic synapses. In the gerbil MSO this time-locked inhibition tunes ITD sensitivity such that the steepest portion of the MSO spike-rate-versus-ITD function falls in the physiological range of ITDs needed for sound localization [18]. The MNTB projection to the LSO provides the inhibitory side of the inhibitory/excitatory computation in the LSO that results in a comparison of sound levels at the two ears [19]. In cats, the effectiveness of inhibition of LSO principal cells is sensitive to the precise synchrony of contralateral inhibition with ipsilateral excitation [20]. The Kv3.3 channels found in high concentrations on the calyces of Held [6] are thought to enable rapid action-potential repolarization without affecting action-potential threshold, rate of rise, magnitude, or refractory-period duration [8]. Those unique features facilitate high-frequency repetitive firing and likely play a key role in the inhibitory/excitatory synchronization [7]. The present results demonstrate the deleterious auditory consequences of loss of functional Kv3.3 channels in human listeners.

Localization of sounds in the horizontal dimension relies on ITDs and ILDs [21,22]; ITDs dominate localization judgments when low-frequency sounds are present, and ILDs are used when sounds are limited to high frequencies [23,24]. Spatial separation of multiple competing sounds aids sound recognition in complex auditory scenes [25,26] and there is evidence that this "spatial release from masking" is dominated by low-frequency ITD cues [27,28]. The ITD thresholds exhibited by most of the affected listeners in the present study approached the maximum ITDs that are naturally experienced given the dimensions of the human head. This suggests that the affected listeners likely would not benefit from the dominant cue for localization and for spatial release from masking. Similarly, the ILD thresholds of affected listeners were quite high, although we did not test at the maximum naturally experienced levels. Although the affected listeners appeared to show fairly normal pure tone detection thresholds in quiet, we infer that they would show substantial impairment in real-world functions of identifying sources of sounds and of understanding speech in the presence of competing voices.
